# Primary malignant melanoma of the trachea: A case report

**DOI:** 10.3892/ol.2014.2782

**Published:** 2014-12-09

**Authors:** HISAO IMAI, YOSHIO KIYOHARA, SHUSUKE YOSHIKAWA, NAO KUSUTANI, AKIRA ONO, TETSUHIKO TAIRA, HIROTSUGU KENMOTSU, HIDEYUKI HARADA, TATEAKI NAITO, HARUYASU MURAKAMI, TAKEHISA SANO, HIROSHI FUJI, MASAHIRO ENDO, TAKASHI NAKAJIMA, TOSHIAKI TAKAHASHI

**Affiliations:** 1 Division of Thoracic Oncology, Shizuoka Cancer Center, Suntou-gun, Shizuoka 411-8777, Japan; 2 Division of Dermatology, Shizuoka Cancer Center, Suntou-gun, Shizuoka 411-8777, Japan; 3 Division of Radiation Oncology, Shizuoka Cancer Center, Suntou-gun, Shizuoka 411-8777, Japan; 4 Department of Respiratory Medicine, Shizuoka City Hospital, Aoi-ku, Shizuoka 420-8630, Japan; 5 Division of Proton Therapy, Shizuoka Cancer Center, Suntou-gun, Shizuoka 411-8777, Japan; 6 Division of Diagnostic Radiology, Shizuoka Cancer Center, Suntou-gun, Shizuoka 411-8777, Japan; 7 Division of Diagnostic Pathology, Shizuoka Cancer Center, Suntou-gun, Shizuoka 411-8777, Japan

**Keywords:** malignant melanoma, trachea, argon plasma coagulation

## Abstract

Primary cancer of the trachea is rare and accounts for only 0.1–0.4% of all newly diagnosed respiratory tract cancers, worldwide. In the present study, a case of primary tracheal malignant melanoma, a particularly rare type of cancer, is reported. A 68-year-old male presented with a cough and bloody sputum. A chest computed tomography scan revealed a 25×20×15-mm tracheal tumor, located immediately above the carina, which reduced the cross-sectional area of the trachea by ~90%. Histopathological analysis of biopsy specimens determined a diagnosis of malignant melanoma. The patient was treated with argon plasma coagulation and chemoradiotherapy, which restored airway patency, however, metastasis was detected in the lungs. The patient refused further treatment and received palliative care. Subsequently, the patient succumbed to the disease within four months. Thus, although primary malignant melanoma of the trachea is extremeley rare, the possibility should be considered during diagnosis.

## Introduction

Primary cancer of the trachea is particularly rare (1) and accounts for only 0.1–0.4% of all newly diagnosed respiratory tract cancers, which corresponds to 2.6 new cases per 1,000,000 individuals, annually, worldwide (2,3). Approximately 75% of these tumors are squamous cell carcinoma or adenoid cystic carcinoma (4). Malignant melanomas occur primarily as skin lesions and account for 2% of all skin tumors, worldwide (5). Although primary malignant melanoma frequently metastasize to the liver, lung, brain, or bone, this type of cancer rarely occurs in parts of the body other than the skin. The most uncommon form of extracutaneous melanoma is primary tracheal melanoma (6–10). In the present study, the case of a patient presenting with malignant melanoma of the trachea is reported to improve the current understanding of this rare disease. Written informed consent was obtained from the patient.

## Case report

**Figure 1:**
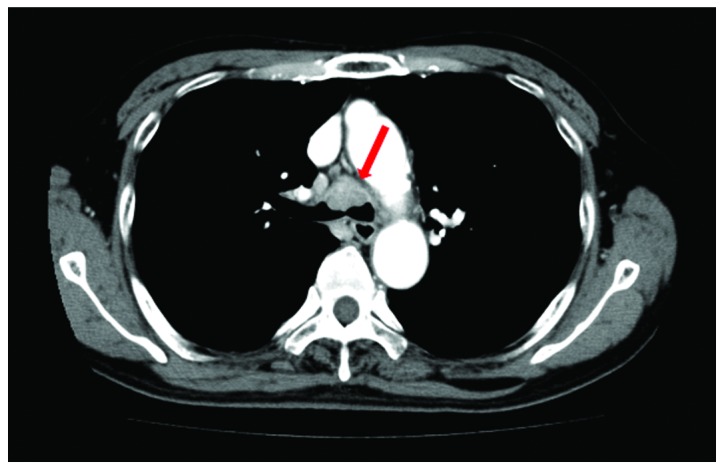
Chest computed tomography scan. The tumor measured 25×20×15 mm and was located directly above the carina and protruded from the anterior wall of the trachea (arrow).

**Figure 2:**
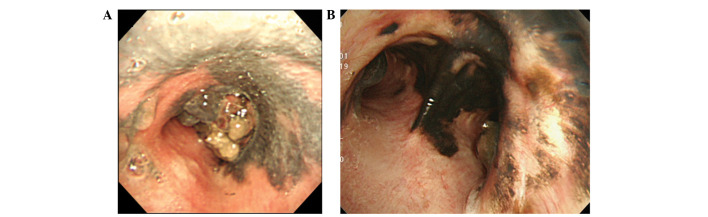
Endoscopic findings. (A) Prior to treatment, bronchoscopy revealed a pigmented, cauliflower-like, warty tumor. (B) Following chemoradiotherapy, bronchoscopy revealed darkened regions in the tracheal mucosa.

In March 2013, a 68-year-old male presented to the Department of Respiratory Medicine, Shizuoka City Hospital (Shizuoka, Japan) with a cough and bloody sputum, which had worsened over the previous two month and become intractable, with the development of stridor. A chest computed tomography (CT) scan revealed a 25×20×15-mm intratracheal lesion located immediately above the carina (Fig. 1), which reduced the cross-sectional area of the trachea by ~90%. Flexible bronchoscopy demonstrated these results and revealed an obstructive tumor surrounding the carina, as well as irregularly shaped, darkened regions in the tracheal mucosa (Fig. 2A).

**Figure 3:**
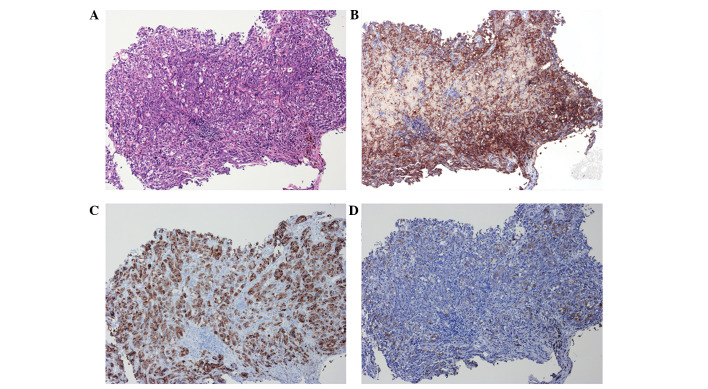
Histological and immunohistochemical observations of tumor tissue obtained via bronchoscopic biopsy. (A) Proliferation of epithelioid-shaped atypical cells with marginal melanin production were observed (stain, hematoxylin and eosin; magnification, ×100). In the same tumor, these epithelioid-shaped atypical cells also stained positively for the melanoma markers (B) S-100, (C) melan-A and (D) HMB-45 (magnification, ×100).

Following biopsy of the tumor and the surrounding mucosa, the tumor was cauterized with argon plasma coagulation (APC) to restore airway patency, however, the presence of a residual tumor mass was not clear. Following cauterization, the patient experienced immediate symptomatic relief. The biopsy specimens were composed of tumor fragments and aggregated melanophages. Histologically, hematoxylin and eosin staining of the tumor demonstrated proliferation of epithelioid-shaped atypical cells with marginal melanin production (Fig. 3A). In addition, the tumor cells showed positive immunostaining for three melanoma markers, S-100, melan-A and HMB-45 (Fig. 3B–D). Subsequently, the tumor was diagnosed as a malignant melanoma. The biopsy specimens from the bronchial mucosa revealed a band-like accumulation of melanophages and lymphocytes beneath the tracheal epithelium. However, melanoma infiltration was not observed among the melanophages.

The patient did not have a history of previous surgeries or skin biopsies and did not exhibit melanoma-like skin lesions. In addition, magnetic resonance imaging and positron emission tomography did not reveal any metastatic lesions in the brain. The level of 5-S-cysteinyldopa, a biochemical marker of melanoma, which was 7.6 nmol/l (normal range, 1.5–8.0 nmol/l) at diagnosis, did not increase. As a result of these findings, it was hypothesized that the trachea was the primary site of the tumor and no metastasis had occurred at the time of clinical presentation.

The lesion was inoperable due to its large size; therefore, the patient was treated with a combination of dacarbazine-based chemotherapy (200 mg/m^2^ dacarbazine, days 1–5) for three cycles every 4 weeks, for three months and thoracic radiotherapy (total dose, 65 Gy in 30 fractions). Following chemoradiotherapy, bronchoscopy revealed darkened regions of the tracheal mucosa (Fig. 2B). Subsequently, metastatic lesions appeared in the lungs and the 5-S-cysteinyldopa levels gradually increased, thus, chemotherapy was resumed.

## Discussion

Primary tracheal malignant melanoma is particularly rare (6–10) and there are only a small number of reports regarding intratracheal metastasis (11). Various studies have investigated the oncogenesis of mucosal melanomas and have attempted to elucidate the histogenesis of lower respiratory tract melanomas (12,13). Theories include melanocytic migration during embryogenesis, transformation of respiratory epithelial cells into melanocytes and differentiation of neuroendocrine cells to melanocyte (12).

Pathological examination cannot distinguish primary melanoma from metastatic melanoma. The criteria for primary respiratory malignant melanoma diagnosis are as follows: A solitary lesion; ‘dropping off’ of melanoma cells together with junctional changes in the mucosa; invasion from the epithelium toward the submucosa; histologically identified presence of melanin; no prior skin lesions; and no familial history of cutaneous disease (12). In the present case, no other primary lesions were identified on radiological or dermatological examination. The patient was diagnosed with a primary tracheal malignant melanoma on the basis of three criteria: The lack of a history of skin lesions and a family history of cancer; the presence of a solitary tumor surrounded by abnormal mucosa; and positive immunostaining for three melanoma markers, S-100, melan-A and HMB-45. These results are consistent with the diagnostic criteria for primary malignant melanoma in the respiratory tract (13,14).

Tracheal tumors may be fatal as they occasionally obstruct the airway. However, due to their rarity, no standard treatment has been identified. Treatment is either palliative, which aims to restore airway patency, or therapeutic, with tracheal resection and end-to-end anastomosis (15). In the current case, APC was conducted to restore airway patency. This indicated that treating tracheal tumors with APC may be an effective type of palliative therapy to provide immediate relief, as well as long-term improvements in patient quality of life. Although it is useful to combine palliative measures with therapeutic agents, including radiation and biological or chemical agents, the current treatment strategies are inadequate. For example, radiotherapy may be an effective method to locally control tracheal melanoma, however, it does not improve long-term survival (16).

In conclusion, the patient in the current case was treated with radiation and dacarbazine-based chemotherapy, which is a standard chemotherapeutic agent for malignant melanoma. Initially, the radiotherapy facilitated with controlling the local spread of the tumor, however, follow-up CT scans revealed distant metastasis to the lungs. In future, targeted cancer therapies using molecules, such as BRAF inhibitors, and cytotoxic T-lymphocyte-associated protein, programmed cell death protein 1 and programmed death-ligand 1 antibodies (17–20), may be effective options for treating cases of advanced malignant melanoma, including those originating in the trachea.
